# Neuroscience of foraging

**DOI:** 10.3389/fnins.2014.00081

**Published:** 2014-04-21

**Authors:** Benjamin Y. Hayden, Mark E. Walton

**Affiliations:** ^1^Department of Brain and Cognitive Sciences and Center for Visual Science, University of RochesterRochester, NY, USA; ^2^Department of Experimental Psychology, University of OxfordOxford, UK

**Keywords:** foraging, foraging theory, intertemporal choice, risk, ecological rationality, diet

The papers that accompany this Research Topic fall at the intersection of foraging theory and neuroscience. Why does such a topic merit a Research Topic in Frontiers in Decision Neuroscience? And what does foraging theory have to do with decision neuroscience?

Foraging theory was created in the 1960's, as behavioral ecologists began to absorb the intellectual advances in microeconomic theory of the 1940's and 50's, and apply its principles to their research. Early foraging theorists realized that animals can be thought of as economic decision-makers that thrive by learning to maximize benefits and minimize costs. A key insight was that adaptive fitness—the main driving influence in Darwinian evolution—behaves mathematically like any other economic good, and that we can analyze behavior by assuming animals seek to maximize it (McNamara and Houston, 1986; Stephens and Krebs, 1986).

While economics focuses on human problems, such selecting a brand of peanut butter or choosing a retirement plan, foraging theory focuses on animal problems. Early foraging theorists identified two major abstract problems: whether to accept or reject a prey item (the diet selection problem) and, when foraging in a prey-rich patch, when to leave it and move on to another one (the patch-leaving problem, Stephens and Krebs, 1986). Of course, these problems apply to humans as well, from the hunter-gatherer foraging for small animals to the internet surfer looking for interesting articles to pass the time.

In the diet selection problem, an animal (e.g., a fox) must decide, on encountering a prey (e.g., a pheasant) whether to pursue it or pass it up (Krebs et al., 1977). Foraging theorists realized that animals should integrate the costs and benefits of pursuit (presumably learned through experience) into a single decision variable and then compare that to specific threshold (also learned). The realization that the optimal strategy is a step-function and the method for computing this threshold were major early discoveries. Importantly, the threshold is a “background variable” that represents the marginal intake rate associated with the overall environment. In economic terms, it is the opportunity cost of pursuit. This example illustrates three key features of foraging problems: (1) they are fundamentally optimization problems that can be solved through cost-benefit analyses, (2) they are modeled after problems encountered by animals, and (3) decisions are usually framed as a foreground (pursue) vs. background (ignore), rather than as two simultaneously presented alternatives as in standard economic tasks.

Several scholars have suggested that foreground-background decisions, even if they are mathematically identical to two-option choices common in economics, are mediated by distinct mental operations (Stephens, 2002). Indeed, it has been suggested that many animal decision-makers have difficulty making two-option choices, and use degenerate strategies evolved to solve foraging problems (Pavlic and Passino, 2010; Kacelnik et al., 2011). Furthermore, there is evidence that human decision-making is framed in terms or a default and an alternative, and that values of these options may be associated with specific brain regions (Kolling et al., 2012; Boorman et al., 2013). These findings highlight the utility of considering foraging-like problems when investigating the mechanisms of economic choice.

Indeed, results obtained in foraging conditions are often different from those obtained in economic tasks. For example, consider intertemporal choice tasks, in which animals choose between a large reward available after a long delay or a smaller reward available sooner. Animals typically reject the larger gains if the delay is more than a few seconds. This seemingly impulsive behavior has often been used to argue that most animal species discount future rewards heavily (Rachlin, 2000; Heilbronner et al., 2008; Kalenscher and Pennartz, 2008; Stevens and Stephens, 2008). Puzzlingly, however, in foraging tasks, animals discount only weakly or not at all (Stephens and Anderson, 2001; Hayden et al., 2011). Some scholars have suggested that the two-option structure of the standard intertemporal choice task is confusing for animals and that, because they misunderstand its structure, it produces highly biased estimates of discounting rates. In contrast, foraging tasks, with their naturally-inspired structure, do not (Bateson and Kacelnik, 1996; Kacelnik, 1997; Stephens, 2002; Pearson et al., 2010; Blanchard et al., 2013; Blanchard and Hayden, 2014).

Another example comes in the context of risky choice. Foraging theory emphasizes the serial and long-term strategic nature of risky choice, and thus suggests that decisions ought to be studied not solely in terms of non-linear utility—in other words, the prospect of an immediate gain or loss multiplied by its likelihood—but in terms of discontinuities between short and long-term strategies (Hayden and Platt, 2007; Heilbronner and Hayden, 2013). Whereas, economic theory often categorically classifies organisms into those that are risk seeking or are risk-averse, studies that look at repeated choices with uncertain outcomes find that both humans and other animals can adapt their choice strategies depending on the state of the environment, their current needs, and their long-term goals (Real and Caraco, 1986; Kolling et al., 2014).

Foraging theory's emphasis on adaptive significance allows us to consider each species within its own ecological niche. For instance, most nutrition a rodent will encounter is at ground level; danger will likely come from above. Rodent superior colliculus (SC) maps precisely this distinction, with visual input from the upper quadrant of the visual field accessing the medial SC, which mediates defense responses, whereas the lower quadrant projects more strongly to lateral SC, stimulation of which results in approach behaviors (Comoli et al., 2012). The manual dexterity and trichromatic binocular vision of primates, and ultimately potentially the development of the prefrontal cortex, can also be related to their need to forage for ripe fruit and tender leaves in a visually complex, cluttered, and volatile environment (Passingham and Wise, 2012). As we neuroscientists try to collate fine-grained research from fruit flies and zebra fish to genetically modified rodents and primates, such ecological considerations will become increasingly pressing.

The papers included in this collection serve as an introduction to some of the major ideas that have influenced the nascent field of neural foraging. The most basic step in deriving a complete understanding of the neural basis of foraging is to understand the neural basis of the building blocks of foraging, food consumption, and executive control of decision-making. One goal is to make foraging theory more biological; whereas we assume that food consumption—often considered simply as “reward” in many neuroscience paradigms—is a “frictionless” process, it is in fact a very real and complex one. To understand it we must understand how it works (Caracheo et al., 2013; Horst and Laubach, 2013), and how it interacts with decision-making (Murray and Rudebeck, 2013). Simultaneously, foraging involves complex cognitive processes, and understanding how those work is critical for understanding the way that our minds constrains foraging decisions (Sallet et al., 2013). These include understanding of the neural representation of variables that are psychologically relevant to foraging decisions, like effort and risk (Miller et al., 2013), as well as social factors (Pearson et al., 2013). Traditional foraging theory tends to ignore factors that affect animal decisions like aging (Mata et al., 2013). Future work will also point toward linking economic ideas to foraging ideas, especially in the domain of time and risk (Bixter and Luhmann, 2013).

## Conflict of interest statement

The authors declare that the research was conducted in the absence of any commercial or financial relationships that could be construed as a potential conflict of interest.
